# Atlanta Academy of Medicine

**Published:** 1873-11

**Authors:** C. A. Simpson

**Affiliations:** Reporter


### Atlanta Academy of Medicine.

C. A. SIMPSON, M. D., Reporter.

April 21st, 1873.

Dr. J. G. Westmoreland, Vice-President, in the chair.

Dr. Anderson reported a case of accidental poisoning with aconite. He was called to a lady with cholera-morbus, and gave a teaspoonful of fluid extract aconite root by mistake for tincture of opium. In a few minutes the usual symptoms of aconite poisoning came on, with tingling sensation in the tongue, tips of the fingers, the toes, etc.

As soon as he discovered the mistake, he administered thirty grains of ipecac with warm water, and repeated the dose at short intervals until she vomited freely. He gave, also, five minims fluid extract gelseminum, one-eighth grain morphia and toddy.

The symptoms of poisoning passed away and she rested well the night. Thinks the cholera-morbus was cured by the aconite.

Dr. Anderson reported, also, a case of uterine polypus. The polypus was removed with the *ecraseur* by Dr. W. F. Westmoreland and himself. Not more than a drachm of blood was lost in the operation. The tumor is in the hands of Dr. Rauschenberg, for microscopical examination.

Dr. Anderson reported, also, a case of senile gangrene of the foot, in a man apparently stout and otherwise healthy.

About a month ago he amputated the distal phalanx of the little toe. In a few days a line of demarcation formed higher up, and he removed two of the toes entire. On yesterday the patient died with symptoms of pyæmia.

Treatment.—Bandage, charcoal poultice, small doses calomel; also, stimulation and nourishing diet.

---

June 2d, 1873.

Dr. J. P. Logan, President, in the chair.

Dr. W. S. Armstrong reported the following case: On Monday he was called to see a boy, seventeen years old, who had gone to the field as usual in the morning, not much complaining.

On examination he discovered a slight papular eruption on the hands and feet, which he thought might have been caused by some poison vine, as he had been cutting in the woods the previous Saturday. There was some febrile excitement; pulse 90 to 100; a simple prescription was made. Seen again on Wednesday. The eruption was no longer papular, but maculated; size of a nickel, to that of the palm of one's hand; of bright red color. It now extended up the legs and arms. He was satisfied that it was not poisoning, and suspected meningitis. There was neither rigidity of muscles nor pain in the cervical spine.

On Thursday there were symptoms of meningitis—delirium the previous night, and some to-day—with dilated pupils. Was now fully convinced of the nature of the malady.

On Friday there was distinctly marked rigidity of the muscles of the neck, and the patient died that night—no autopsy.

The Dr. called attention to the papular eruption as a premonitory symptom in this case, and inquired if other members of the Academy had observed similar cases.

The President, Dr. Logan, had seen purpuric eruptions in meningitis, but had not observed the papular eruption described by Dr. Armstrong.

Dr. Taliaferro thought it a matter of importance, both for the Academy and the profession generally, that notice be taken of these eruptive manifestations in connection with meningitis, in consequence of their bearing upon the hypothesis of a peculiar blood-poisoning.

Dr. Rauschenberg inquired if there was any extravasation.

Dr. Armstrong replied, he thought there was from the appearance of the spots.

Dr. Rauschenberg thought if these maculæ were extravasated blood, it might be caused by some abnormal state of the capillary circulation—either due to passive congestion, or to a pathological condition of the coats of the vessels, or the blood itself, dependent upon deficient action of certain nerve-fibres. Had met frequently with such local effusions, limited to certain capillaries.

Dr. Logan desired to call the attention of the Academy, to a subject brought before the American Medical Association, at its recent meeting in St. Louis. A very interesting paper, on the subject of cancer, was read by Dr. Woodward, who, after a very close investigation of the subject, arrived at the conclusion, that cancer does not originate in the blood, but is altogether local in its origin. He therefore attaches very great importance to the early extirpation of cancerous growths.

This opinion of Dr. Woodward is of special interest, in as much as it is at variance from the generally received opinion of the profession. Would simply call attention to this one point, as the paper itself will soon be published.

Dr. Rauschenberg stated, that this had been for sometime the received opinion among many of the medical profession of Germany.

---

JUNE 9TH, 1873.

Dr. J. G. Westmoreland, Vice-President, in the chair—

Dr. J. G. Westmoreland reported the following case:

Called Friday 9 A.M., to child, aged 3 years and 10 months. He had been extremely restles for several hours, and had slept but little. There was much nervous agitation—pulse small and frequent—cervical muscles perfectly rigid. By putting the hand under the head, could raise the body up without bending the neck. There was anxious expression and evidence of great suffering.

Diagnosed meningitis, and began active treatment at once. The pulse was soon reduced in frequency, with improved volume. There was no longer muscular spasm or nervous agitation, and the chin could be approximated to the chest, but upon removing the hand, it would fly back like india-rubber.

In the evening he was surprised to find the patient even worse than at the first visit. The pulse was again very frequent and small, and every effort to improve the condition was unavailing. During the night and Saturday there was no amendment, and he died Saturday night. No autopsy.

DIAGNOSIS.—Meningitis, with cerebritis.

TREATMENT.—Venesection, cups, and mercury. Bled promptly from the arm four ounces; cupped one ounce from back of neck. Friday night, cupped one ounce more.

Dr. Rauschenberg inquired how long before the pulse came down?

Answer—A few hours after depletion.

Dr. R. has frequently seen this change in the pulse without any depletion. In one case there were for some weeks distinct intermission, and then an aggravation of the symptoms. Thinks these intermissions a most serious objection to the theory of the inflammatory nature of meningitis. Most prominent authors describe these intermissions and remissions. Kleinod, of New York, speaks of this among the symptoms; also, Neimeyer and Hirsch, of Germany. Has noticed intermission of pulse as one of the symptoms in almost all cases. Saw a case last year, with Dr. Owen, which had all the symptoms of meningitis, in which this unsteadiness of the pulse was particularly well-marked for six weeks, there being remissions every morning. Holds that true inflammation is regular and unchangeable in its course, and not intermittent, as in these cases.

Dr. W. replied, that his case did not pursue the course of that referred to by Dr. R. There was only one intermission, in which all the grave symptoms of spasm, agitation, etc., subsided. These symptoms gradually returned, and there was no other intermission.

Dr. Baird asked why he suspected inflammation of the substance of the brain.

Dr. W.—Because of the violence of the symptoms and character of the delirium. He was not comatose; could be aroused with some difficulty, but did not seem to know anything; if anything was put into the mouth, would swallow mechanically; did not appear to be any pressure upon the brain. Vomiting was a prominent symptom; pupil contracted; never saw this before—usually dilated; there was slight movement of pupil under strong light; after treatment, pupils were of normal size.

Dr. R. never saw the bounding pulse of inflammation in meningitis,—never heard of a truly inflammatory pulse in these cases; considers a weak and variable pulse characteristic of the disease, and thinks he could recognize it by this and the rigidity. He is firmly convinced that the pulse in meningitis is different from the inflammatory pulse. There is certain to be more or less intermission, sooner or later, in the disease. According to later opinions, any acute change in the nutrition of the capillaries is called inflammation. These changes depend upon many different conditions; by whatever condition this disturbance may be occasioned, does not matter. Virchow speaks of inflammation when there are no blood-vessels. If these changes in nutrition take place in this disease, will admit that it is inflammatory.

Dr. W. has noticed the small pulse in cases of pneumonia, and, in his own case, in acute pleuritis. In these cases the pulse may be small during the whole course of the disease. Would mention a case of nephritis, seen with Dr. Connally, in which the pulse was steady and firm for six weeks, with not much variation. There has been vomiting in almost all the cases of meningitis he has seen.

Dr. Baird has noticed a small and irregular pulse in all cases he has seen. Diagnosed one case by this symptom alone; vomiting always prominent.

Dr. J. T. Johnson agreed with Dr. B. as to the irregularity of the pulse,—has noticed it in all his cases; vomiting not always present; temperature is high, especially in the latter stage.

